# Slip-Resistant Connections with Hot-Dip Galvanized Faying Surface Under Freeze-Thaw Cycles and/or Low Temperature

**DOI:** 10.3390/ma18010084

**Published:** 2024-12-28

**Authors:** Alfonso Fuente García, Miguel Serrano López, Carlos López-Colina Pérez, Fernando López Gayarre

**Affiliations:** Polytechnic School of Engineering, Campus de Viesques, University of Oviedo, 33203 Gijón, Spain; serrano@uniovi.es (M.S.L.); lopezpcarlos@uniovi.es (C.L.-C.P.); gayarre@uniovi.es (F.L.G.)

**Keywords:** slip resistant connections, freeze-thaw cycles, low temperature, hot-dip galvanized, faying surface, preloaded bolts

## Abstract

In some occasions, outdoor steel structures like wind towers, bridges, winter sports facilities, and so on are subjected to extreme environmental conditions with the presence of ice and/or with below-zero temperatures. Sometimes in these situations, surface protection of the steel structure is usually designed using hot-dip galvanizing to improve its durability. In these special circumstances, the structure’s connections are also exposed to adverse climatic agents. International standards and codes such as Eurocode 3 or EN1090-2 do not provide indications for these cases. In this experimental research, 24 specimens of non-slip joints with hot-dip galvanized faying surfaces and HV M16 and M20 bolts have been studied. Twelve specimens were subjected to fourteen twelve-hour freeze-thaw cycles, with temperature oscillation and periodic immersion in water. Next, six of the connections were subjected to a slip test under monotonic load at a temperature of −20 ± 0.5 °C and the other six at room temperature. The results were compared with joints kept at room temperature and not subjected to freeze-thaw cycles for the same period of time. The main conclusion of this piece of research is that the short-term slip resistance behavior of joints with hot-dip galvanized surfaces is not reduced for the cases studied.

## 1. Introduction

When a steel structure is subjected to vibratory, dynamic loads or with changes in direction loads caused by cyclic, seismic, impact, or wind forces, the use of slip-resistant connections should be considered to improve service conditions and reduce local deformations. Specifically, European standard Eurocode 3 Part 1.8 [1] establishes that slip-resistant pre-loaded bolted joints must be used when slip of connection is not acceptable. This code defines in section 3.4.2, five categories of connections, three of them for shear forces named A, B, and C, and the other two, D and E, for tension loads. Each type of connection must be verified with a specific criterion in order to check it. Slip-resistant bolted connections are considered category B when they are designed for serviceability limit state or category C when the design is at ultimate limit state. For both cases, the preload applied to the bolt generates a compression force between contact surfaces, creating a friction resistance that avoids the relative movement of plates. This means that the shear force is not withstood by the bolt shank but by the friction force between the surfaces. Therefore, it is not necessary to check the bolt shank for shear load.

Outdoor steel structures are sometimes subjected to extreme environmental conditions with the presence of ice and/or with below-zero temperatures. Some examples of these types of structures are steel bridges, winter sports facilities, ski lifts, or mining transfer towers. Temperature variations in profiles are widely analyzed in international codes and standards; even the accumulation of ice on them is included in some, such as ISO 12494 [2], EN 1993-3-1 [3], or EN 1993-3-2 [4]. However, not much literature has been found that investigates the influence that these events can produce in profile joints and, more specifically, in slip-resistant connections, with the exception of some such as the German code VDI2230 [5], which provides information on the variation in the bolt preload due to temperature, or others such as those performed by Ebert et al. [6], which warn about the loss of preload of joints in outdoor structures over time.

One of the best solutions usually used to protect the surfaces of steel profiles from corrosion due to the presence of ice and/or humidity is hot-dip galvanizing (HDG). Hot-dip galvanizing generates a complete coverage and a uniform protection on the steel surface that has several benefits like longevity, durability, sustainability, and reduced life-cycle costs. When choosing to use an HDG as a surface protection system, slip-resistant connections cannot be easily isolated from the whole surface treatment due to the hot-dip process. It means that the slip coefficient values specific to the hot-dip galvanized surface must be considered to calculate their slip resistance. In this sense, it is interesting to note how the latest version of the EN 1090-2 standard of 2019 [7] includes in Table 17 the slip factor data for hot-dip galvanized friction surfaces, which did not exist in the previous version of 2008 [8].

Taking into account all the above, it is worthy to further understand whether non-slip joints with HDG surfaces are affected in any way by being exposed to freeze-thaw cycles and/or low temperatures. This present study is in continuity with the previous work carried out by the current authors [9,10] about slip-resistance with different types under extreme conditions of low temperature and freeze-thaw cycles. The novel contribution and the main innovation of this paper is to test the reliability of the slip-resistant connections with hot-dip galvanized surfaces under extreme conditions such as freezing and thawing in the presence of water and/or at low temperatures. Previous similar studies did not include this type of surface in their analyses.

The objective of this research is to try to fill the detective gap by providing how freeze-thaw cycles and low temperature, separately and jointly, affect slip-resistant bolted connections with M16 and M20 (10.9), steel plates S275, and grit-blasted Sa 2½ before hot-dip galvanizing the faying surface (GB + ZN). The findings provide assurance regarding the use of this type of non-slip surface under the extreme environmental conditions indicated.

## 2. Bolted Slip-Resistant Joints

### 2.1. Slip-Resistance Capacity

Bolted slip-resistant joints use friction between surfaces to limit the relative sliding of the joined parts. There are two factors that influence the slip resistance capacity of the joint. On one hand, we have the roughness of the faying surfaces, which will depend on the surface finish of the parts, and on the other hand, the clamping force between the parts, which will depend on the tightening applied to the bolt. The tightening produces an axial preload on the bolt and consequently a compression force between the plates, generating a resistance to sliding, which, according to section 3.9.1 of Eurocode 3, must be calculated with the Equation (1).

(1) $$F_{s,Rd}=\frac{k_{s}\cdot n\cdot \mu }{\gamma_{M3}}F_{p,C},$$

where *k*_*s*_ is a parameter of hole type (*k_s_* = 1 for normalized round hole); *n* number of friction planes, *μ* the slip factor obtained by specific test for the friction surface, *γ*_*M*3_ partial safety factor, and *F*_*p*,*C*_ the preload force in the bolt.

### 2.2. Bolt Preload

It is commonly accepted that a preload value of 70% of the yield strength of the steel is used in the high-strength bolt. This ensures that the bolt does not work in the plastic zone. The preload on the high-strength bolt can be applied by different methods, although the EN 1090-2 standard allows the following four types: torque method, combined method, HRC method, and direct tension indicator (DTI) method. To estimate the preload to be applied to the bolt, Eurocode 3 provides the Equation (2).

(2) $$F_{p,C}=0.7\cdot f_{ub}\cdot A_{s},$$

where *f_ub_* is the ultimate tensile strength of the bolt and *A_s_* the net cross section of the bolt in the threaded zone.

In European regulations, bolts suitable for preload and use in steel structures are specified in Eurocode 3 and in EN 1090, and must follow the requirements, dimensions, and characteristics included in the EN 14399 set of standards. The bolt, nut, and washer assemblies are usually more robust than those for conventional bolts and must have sufficient capacity to maintain the preload during their useful life.

It should be noted that the EN 14399 [11] standard defines two types of assemblies for slip-resistant joints with different dimensions and resistant sections. On one hand, there is the German environment system HV (Hochfest Vorspannbar), and on the other; the UK environment system called HR (high resistance), both composed of a bolt, a nut, and two washers. Regarding the type of steel, the HV system allows the use of class 10.9, while the HR allows the use of classes 8.8 and 10.9.

According to all of the above, Table 1 resumes theoretical preload force for M16 and M20 HV bolts with class 10.9 and dimensions corresponding to EN 14399-4 [12].

### 2.3. Faying Surface

As indicated before, the type of faying surface is the other parameter necessary to determine the slip resistance capacity of a slip-resistant connection *Fs*,*Rd*. According to different standards, the coefficient of friction *μ* must be obtained empirically for the contact surfaces existing in the project; however, they usually provide estimated values for common surfaces that the designer of steel structure joints can use directly. In the European environment, the latest version of EN 1090-2 includes in its section 8.4 the slip factors shown in the following Table 2.

For the US environment, the RCSC [13] code provides in section 5.4 slip factor values for different surfaces shown in Table 3.

Note that both regulations provide non-identical values for the same type of surface, taking into account that the tests are quite different. These differences were also noted for other international standards, such as the Australian AS 4100 [14], the Japanese JASS6 4.10 [15], or the British BS 4604-1 [16]. At this point, it is worth highlighting the studies carried out by Stranghöner [17,18,19], in which she warns of the multiple factors that can influence the static slip factor for practically the same surfaces depending on whether one slip test or another is considered.

## 3. Experimental Study

### 3.1. Test Programme

To implement the experimental program, bolted connections with the following features and conditions were considered:

- Bolts: M20 and M16 (10.9) HV (EN 14399)
- Assembly steel plates S275.
- Faying surface: Grit Blasted Sa 2½ (GB) and subsequent Hot-Dip Galvanized (ZN)
- Freeze-thaw cycles before the slip tests: 14 cycles of 12 h.
- Slip test temperature: +20 ± 1 °C (RT) and −20 ± 0.5 °C (LT).
- Slip test speed rate: 0.1 mm/min
- Room relative humidity: 70%

**Figure 1:**
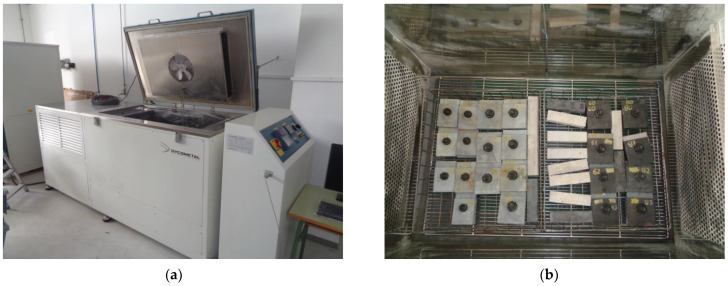
Climatic test chamber: (**a**) Model CHD 525 (Dycometal, Barcelona, Spain); (**b**) Samples set in a climatic test chamber (left ones).

As indicated before, this piece of research requires exposing half of the specimens to 14 freeze-thaw cycles of 12 h each in a climatic test chamber shown in Figure 1. The small size of the internal dimensions of this climatic chamber did not allow the use of the specimens defined in the EN 1090 slip test.

**Figure 2:**
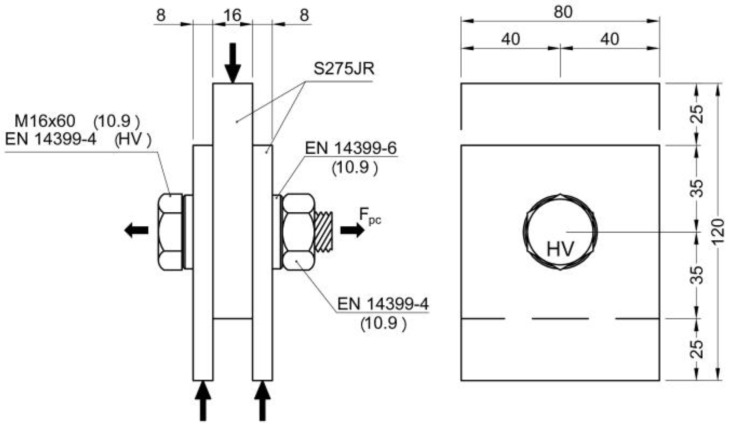
Description and dimensions of M16 specimens.

**Figure 3:**
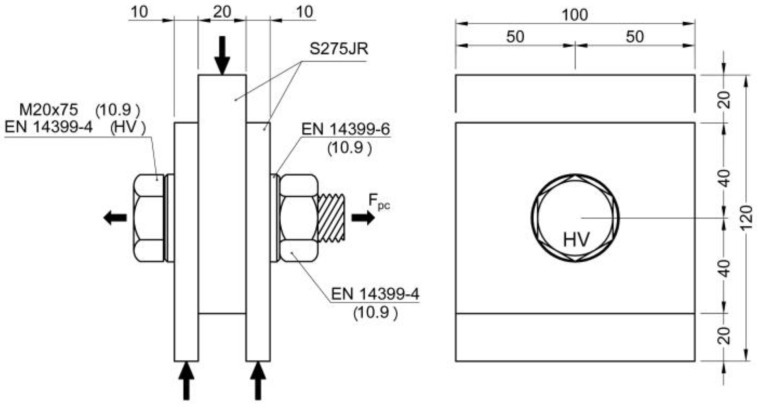
Description and dimensions of M20 specimens.

For this reason, it was decided to use single-bolt samples and perform compression slip tests in a similar way to the RCSC test with the plate thicknesses, bolt diameters, and steel grades of European standards. The dimensions of the M16 and M20 samples are shown in Figure 2 and Figure 3, respectively. The clearance of the holes for inserting the bolts was 2 mm in both cases.

**Figure 4:**
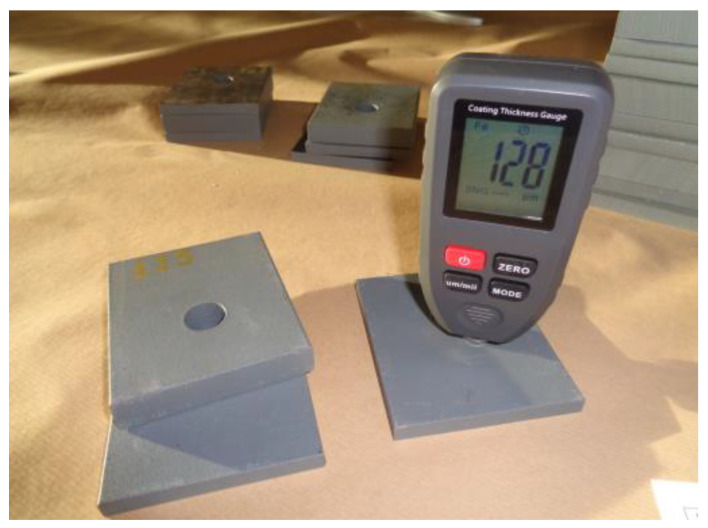
Coating thickness measurement.

All samples were subjected to grit blasting with triangular cast steel particles with size LG-40 according to grit size specification SAE J444 [20]. This allows achieving a cleaning grade of Sa 2½ (very thorough blast-cleaning) according to the generalized criteria of Swedish code SIS 055900 [21]. Notice that this standard of surface preparation has been assumed by the international specification ISO 8501-1 [22]. Grit blasting is indicated for a deep abrasive cleaning surface, which facilitates good zinc fixation during the hot-dip galvanizing process that is carried out subsequently. Since the plates used in the samples are less than 15 cm in size, galvanization in a conventional electrolytic tank is not recommended. This is because during the handling and fixing of the pieces, nodules, blisters, roughness, and sharp points often occur, producing a heterogeneous final result. So, in this experimental study, a centrifugal galvanization system was used, in which the pieces were placed in metal or perforated baskets and centrifuged after being removed from the zinc bath. This allows the excess zinc to be removed and a uniform thickness and a more homogeneous final appearance to be achieved. To check the coating obtained, the thicknesses were measured with a Telam TC-100 (R&D, Shenzhen, China) thickness gauge as shown in Figure 4.

Hot-dip galvanizing was performed in accordance with EN ISO 1461 [23], obtaining an average thickness of the galvanized coating of 97.7 microns, which is slightly higher than the minimum value of 85 μm recommended by the code. The values obtained are shown in Table 4.

Two series with identical preparation were compared: one was subjected to freeze-thaw cycles in a climatic test chamber, while the other was kept at room temperature for the same duration. Following this, all samples underwent a short-term slip test using a universal testing machine. Table 5 summarizes the tested series. Three tests were performed for each situation.

### 3.2. Bolt Tightening Method

**Figure 5:**
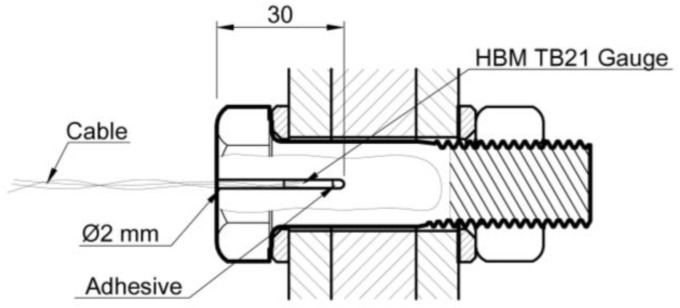
Detail of strain gauge placement inside the bolts.

In this study, the tightening method used was the torque method, applied with a pre-calibrated torque wrench. To determine the exact relationship between applied torque and bolt preload, longitudinal strain gauges TB21 (HBM GmbH, Darmstadt, Germany) were installed on two M16 bolts and another two on M20 bolts, which were previously drilled to insert them. Figure 5 shows the detail of the strain gauges set. The calibration process was carried out on a universal testing machine, measuring the charge–discharge values three times.

**Figure 6:**
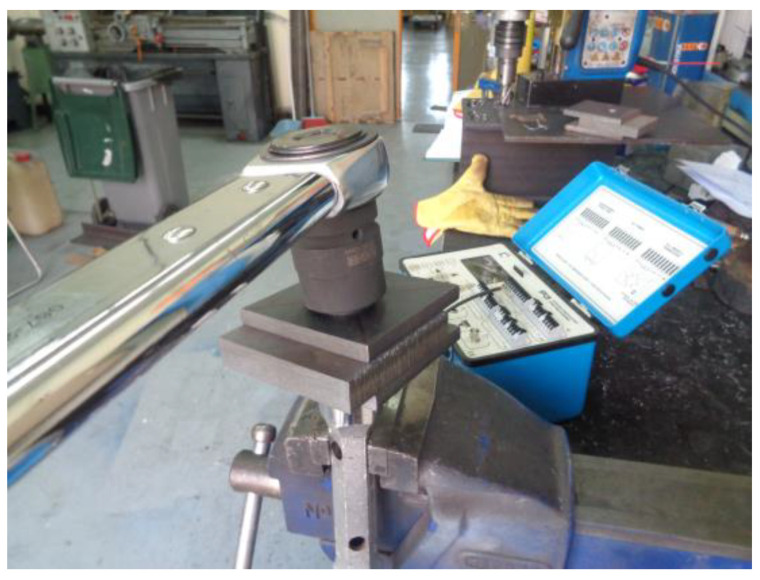
Strain gauge calibration with torque wrench.

The wrench jump values were then adjusted to the average nominal tension values shown on the strain gauges. The final torque wrench values obtained were 320 N∙m for M16 bolts and 615 N∙m for M20 bolts. Prior to applying preload to all specimens, it was confirmed that all bolts and nuts were sourced from the same manufacturer and had the same lubrication. The following Figure 6 shows how the trigger wrench calibration process was performed.

### 3.3. Freeze-Thaw Cycles

After the samples were assembled and preloaded, the 24, 34, 44, and 64 series were exposed to freeze-thaw cycles in the climatic chamber as shown in Figure 1.

**Figure 7:**
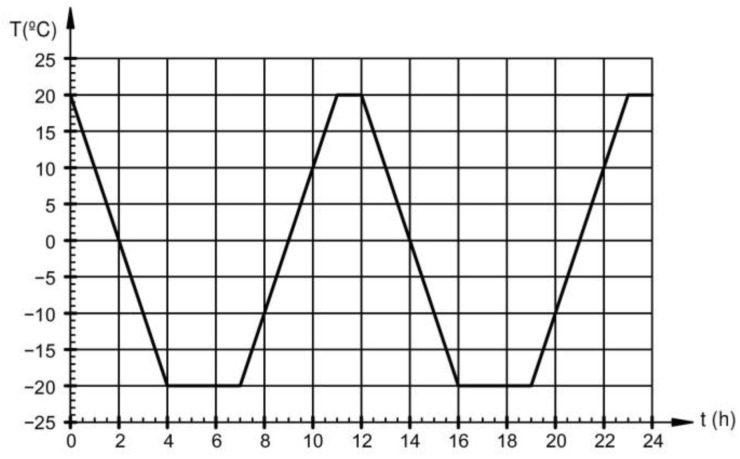
Temperature curve according to UNE-CENTS 12390-9EX.

Since there is no specific standard reference for conducting freeze-thaw cycle tests for outdoor steel structures, the UNE-CENTS 12390-9EX [24] standard for outdoor concrete structures was adopted. This code outlines a temperature curve designed to simulate extreme environmental conditions that structural components might encounter. The samples underwent 14 freeze-thaw cycles, each lasting 12 h, following the temperature curve shown in Figure 7. The temperature inside the chamber fluctuates between 20 °C and −20 °C, with a gradient designed to take 4 h to complete each transition. Once the temperature reaches −20 °C, it is maintained for 3 h, whereas at +20 °C, it is held for only 1 h. The heating and cooling processes are managed by circulating water.

### 3.4. Joint Slip Test

**Figure 8:**
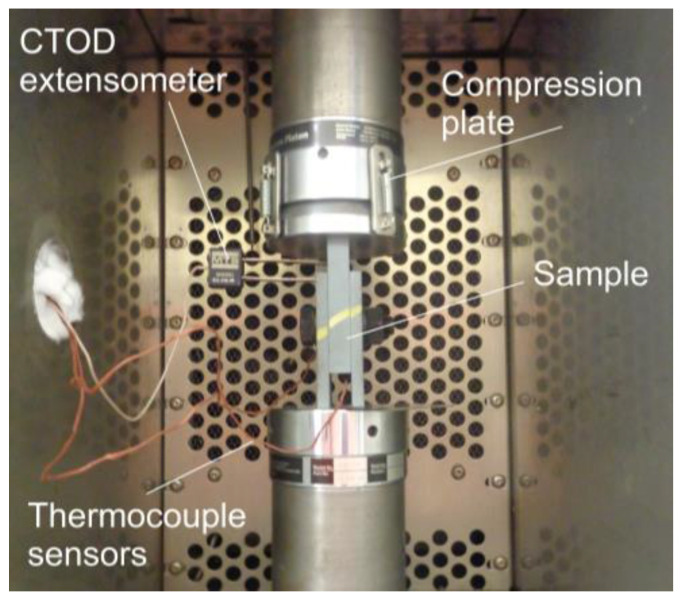
Slip and temperature measurement.

The joint slip test was performed on an MTS 312 (MTS Systems Corporation, Minneapolis, MN, USA) universal testing machine with a load capacity of up to 250 kN. A quasi-static short-time slip test was performed at a feed rate of 0.10 mm/min, applying an incremental compressive force under displacement control. To record the relative displacement between joint plates, a CTOD (crack tip opening displacement) extensometer was used as shown in Figure 8. A pair of plates with spherical coupling was utilized to eliminate eccentricities during the testing process. The tests were concluded when the slip reached 0.50 mm. The force-slip curves for each situation were obtained as the average of the data obtained for three tests.

**Figure 9:**
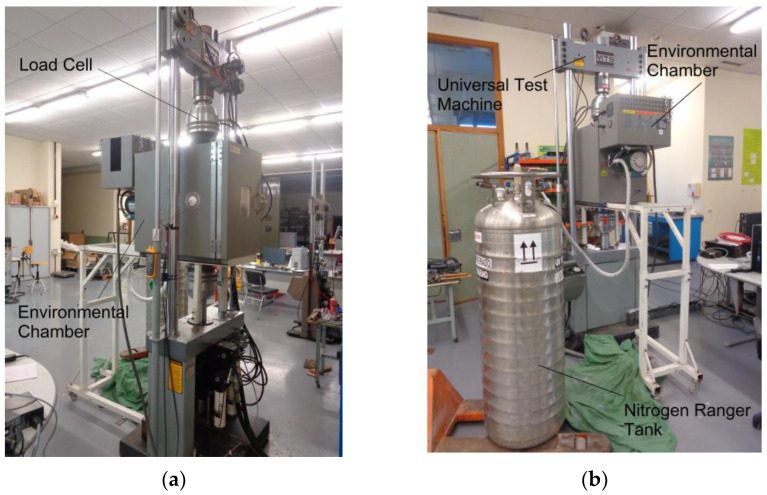
Low temperature testing set: (**a**) Front view; (**b**) Rear view with nitrogen liquid ranger.

The slip test for series 34, 44, 74, and 84 was carried out at a low temperature of −20 ± 0.5 °C. This required coupling an ad hoc climatic chamber to the universal testing machine to maintain the samples at a low temperature throughout the test. An MTS-651.06 E-03 (MTS Systems Corporation, Eden Prairie, MN, USA) environmental chamber with upper and lower openings was used, enabling the insertion of two cylinders specifically machined for these types of testing. A small supporting structure was required to hold the chamber in place. Cooling was provided via liquid nitrogen from a ranger tank that was directly connected to the environmental chamber as shown in Figure 9.

The slip test of series 14, 24, 54, and 64 was performed at room temperature (20 ± 0.5 °C), keeping the rest of the conditions the same as the low-temperature series.

## 4. Results

### 4.1. Slip-Load Curves

**Figure 10:**
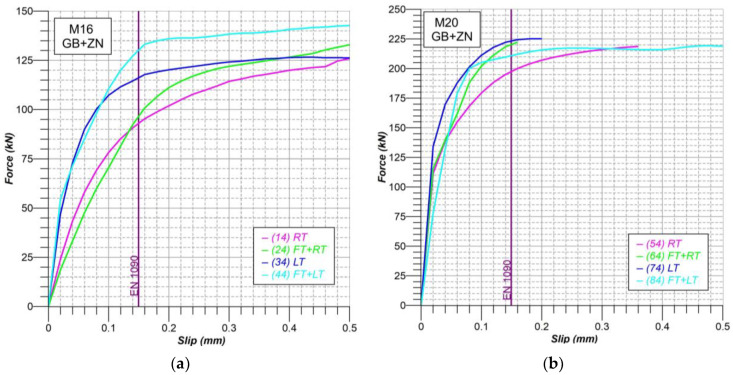
Load-slip curves comparative: (**a**) M16 samples (Series 14-24-34-44); (**b**) M20 samples (Series 54-64-74-84).

The curves obtained for the GB + ZN surfaces showed uniformity and continuity throughout the range studied from 0.00 mm to 0.50 mm. However, their resistant capacity continued to increase significantly even for slip values nearing 0.50 mm, to such an extent that in series 54 and 64, the maximum capacity of the universal testing machine was reached, so these tests had to be stopped at slip values lower than 0.20 mm. As can be seen in the following Figure 10, values close to 230 kN were reached for the M20 samples.

Although the theoretical capacity of the MTS-661 (Lebow Associated Inc, Minneapolis, MN, USA) load cell is 250 kN, it should be noted that the pump that supplies the hydraulic circuit has a slightly lower capacity that is conditioned by other factors such as the temperature of the oil or the ambient temperature of the laboratory.

### 4.2. Slip Factors

The average force-slip curves shown previously allow a graphical visualization of the influence of each of the environmental situations considered on the analysis surfaces and serve as a reference for proposing a behavior curve. However, in order to concretely quantify the influence of low temperature and freeze-thaw cycles on the slip coefficient, it was necessary to establish a reference value for slip that would allow numerical comparison. To do this, the use of the criterion included in standard EN 1090 was considered with a threshold slip of 0.15 mm, calculated according to Equation (3).

(3) $$\mu_{i}=\frac{F_{Si}}{2\cdot F_{p,C}},$$

where *μ_i_* is the slip factor; *F_Si_* the average force at 0.15 mm slip or the peak force before 0.15 mm, and *F*_*p*,*C*_ the preload force in the bolt at the start of the test.

Considering all these factors, the average slip coefficients for the four situations were obtained. The following Table 6 shows the values obtained for the M16 and M20 samples. It should be noted that the possible variations of what are called slip coefficients here are influenced not only by the changes that may occur in the coefficient of friction or rubbing between pieces due to temperature, the presence of ice, humidity, or surface deterioration.

### 4.3. Water Infiltration

**Figure 11:**
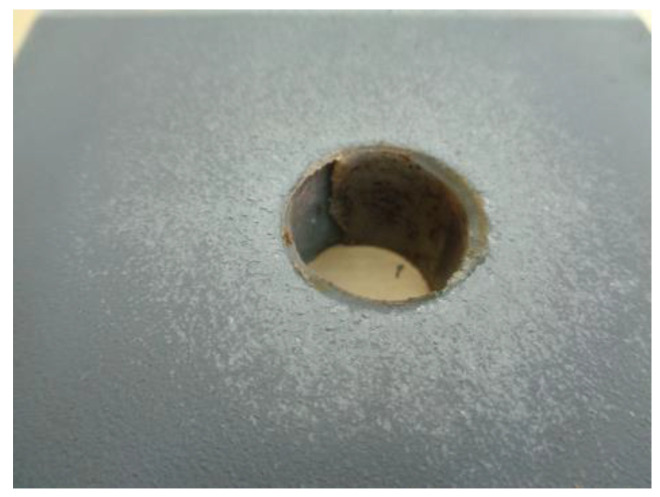
Ice around the hole.

Apart from the quantitative study shown above, the tests showed a relevant circumstance, which was the detection of water inside all the samples previously subjected to freeze-thaw (series 24-44-64-84) once they were unbolted. In the cases of joints exposed to freeze-thaw cycles and tested at low temperature (series 44 and 84), the presence of ice was also confirmed in the entire space between the hole in the plates and the bolt (Figure 11), incorporating ice crystals in the friction zone.

**Figure 12:**
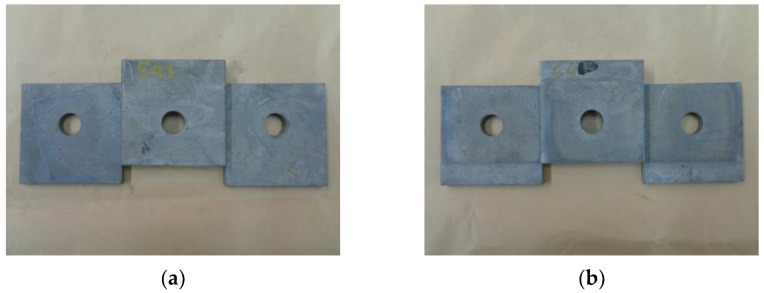

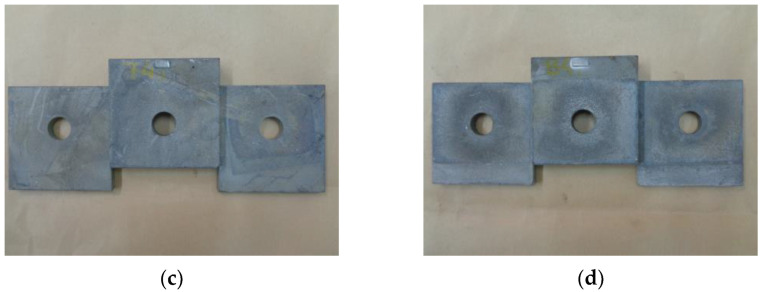
Visual comparison of GB + ZN surfaces after different situations: (**a**) RT samples (Series 14 and 54); (**b**) FT + RT samples (Series 24 and 64); (**c**) LT samples (Series 34 and 74); (**d**) FT + LT samples (Series 44 and 84).

Contrary to what has been shown in other previous studies [9,10] carried out under similar conditions but with other surface finishes, the GB + ZN surfaces studied in this case did not show significant deterioration. Only a slight detachment of small particles in the form of grey powder was observed. It was detected that these particles were from the zinc treatment and that no oxidized or degraded areas were generated. To the touch, the surface appeared intact, maintaining its roughness, and without any noticeable differences with respect to the 54 and 74 series. Figure 12 shows that there are hardly any visual differences between the series that were subjected to FT cycles and those that were not.

## 5. Discussion

Firstly, it is important to highlight that the empirical slip coefficient values obtained for the galvanized surface under normal conditions cannot be directly compared with those provided in EN 1090-2 or RCSC, since the abrasive grit blast cleaning process was carried out before the galvanization, not afterwards as proposed by both codes. In any case, it should be noted that the results presented in this study cannot be directly compared with those given in EN 1090-2 and RCSC due to differences in the samples and the test procedures.

In any case, it should be taken into account that the results presented in this study cannot be directly compared to those provided in EN 1090-2 and RCSC due to differences in the samples and testing procedures.

When comparing the results of tests conducted at room temperature (series 14 and 54) with those of samples subjected to freeze-thaw cycles beforehand (series 24 and 64), a general increase in the slip coefficient is evident for the freeze-thaw treated samples. This increase is observed in both the M16 (series 14) and M20 (series 54) samples, with the M20 samples showing a more noticeable rise of 11.92%. It is interpreted that in these cases, the detachment of metallic particles of the surfaces causes an increase in the friction between the plates.

A comparison of the sliding test results carried out at low temperatures (series 34 and 74) with those performed at room temperature (series 14 and 54) shows, in all cases, an increase in the effective sliding coefficient for joints exposed to low temperatures. However, this increase is found to be uneven depending on the bolt size used. The observed increase in slip coefficient values could be attributed to the rise in the prestressing force of the bolt caused by thermal contraction due to the temperature reduction. However, as demonstrated by A. Fuente et al. [9], for joints with screws and plates of equal dimensions, this effect would not exceed 2%. This suggests that, as previous research has shown [25,26], changes in relative humidity associated with temperature variations and the replacement of the air inside the environmental chamber with dry nitrogen during testing lead to an increase in friction between the contact surfaces.

**Figure 13:**
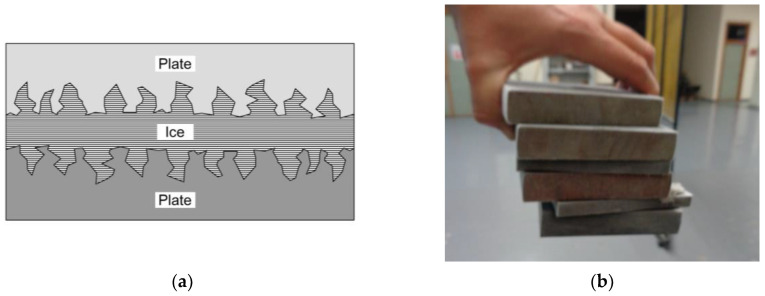
Ice adhesion: (**a**) Adhesion mechanism; (**b**) Ice adhesion capacity between plates.

Comparing the effective results of the samples tested at room temperature (series 14 and 54) with those subjected to the freeze-thaw process and subsequently tested at low temperature (series 44 and 84), we found significant increases of up to 39.45% in the M16 samples. This is mainly due to the fact that the interstitial ice generated inside the samples promotes the connection between the plates through an adhesion process that ultimately improves the sliding resistance of the joint. Previous research, such as that carried out by Loganina and Makkonen [27,28], already demonstrated the adhesion capacity of ice on surfaces in contact. The following Figure 13 shows the adhesion mechanism and the ice adhesion capacity for several non-bolted plates immediately after being removed from the environmental chamber. This mechanism of ice adhesion on surfaces was also analyzed in existing literature by some investigations, such as those carried out by Luca Stendardo et al. [29], Kirill A. Emelyanenko et al. [30], or Monika Bleszynski et al. [31].

By comparing the results from studies conducted by A. Fuente [9,10] on other common faying surfaces like surface as rolled (SR), grit blasted Sa 2 ½ (GB), grit blasted and painted with zinc epoxy (GB + ZE), or grit blasted and painted with inorganic zinc silicate (GB + SL), it is concluded that the most effective solution for slip-resistant connections in steel structures exposed to freezing and thawing is grit-blasted surfaces with hot-dip galvanized.

The standard deviation of the obtained resistant force values was found to be less than 15% in all cases, which strengthens the interpretation of the findings.

## 6. Conclusions

From the findings presented in this study, the following conclusions can be drawn:

1. The effects of freeze-thaw cycles and low temperatures must be analyzed independently, and the effects of both cannot be added algebraically.
2. The slip-resistant joints subjected to freeze-thaw and tested at room temperature (series 24 and 64) show increases compared to those not subjected to freeze-thaw cycles (series 14 and 54). This was clear in both the M16 and M20 samples; however, it is clearer in the latter with an increase of 11.92%.
3. The comparison of the results of the sliding tests carried out at low temperatures (series 34 and 74) with those carried out at room temperature (series 14 and 54) indicates, in all cases, an increase in the effective sliding coefficient for those joints subjected to low temperatures.
4. Comparing the results of the samples tested at room temperature (series 14 and 54) with those subjected to the freeze-thaw process and subsequently tested at low temperature (series 44 and 84), the effective and corrected slip coefficients are increased to a greater or lesser extent, with increases of up to 39.45% observed in the M16 samples.
5. All samples showed slight release of zinc particles in the form of dust; however, the protection of the steel against oxidation was maintained.

In conclusion, it can be affirmed that slip-resistant connections with hot-dip galvanized faying surfaces, when subjected to freeze-thaw cycles and/or low temperatures, demonstrate reliable performance in terms of their resistance under these conditions. Furthermore, the surfaces exhibit minimal deterioration, making this approach a suitable solution for outdoor structures exposed to harsh environmental conditions. It should be noted that the long-term behavior of the connection was not investigated, which directly impacts the final slip factor. The long-term creep behavior of the connection and the effects of freeze-thaw cycles on preload loss over time could affect the reliability of these types of joints.

Since the European reference standards EC3 and EN 1090 do not provide specific requirements for slip-resistant joints under these conditions, it is proposed to incorporate in future revisions the recommendation to use grit-blasted and subsequently galvanized contact surfaces as the most suitable solution. While this treatment requires the full galvanization of the structure, which comes with associated costs, it is important to note that this protection system is already widely used in many metal structures directly exposed to freezing conditions and/or ice.

## Figures and Tables

**Table 1:** Theoretical preload for M16 and M20 bolts (10.9) (HV).

Bolt	*f_ub_*	*A_s_*	*F_p,C_*
M16	1000 N/mm^2^	157 mm^2^	109.90 kN
M20	1000 N/mm^2^	245 mm^2^	171.50 kN

**Table 2:** Classifications that may be assumed for friction surfaces according to EN 1090-2 (2018).

Surface Treatment	Class	Slip Factor *µ*
Surfaces blasted with shot or grit with loose rust removed, not pitted.	A	0.50
Surfaces hot-dip galvanized to EN ISO 1461 and flash (sweep) blasted and with alkali-zinc silicate paint with a nominal thickness of 60 μm.	B	0.40
Surfaces blasted with shot or grit: (a) coated with alkali-zinc silicate paint with a nominal thickness of 60 μm; (b) thermally sprayed with aluminum or zinc or a combination of both to a nominal thickness not exceeding 80 μm.	B	0.40
Surfaces hot-dip galvanized to EN ISO 1461 and flash (sweep) blasted (or equivalent abrasion method)	C	0.35
Surfaces cleaned by wire-brushing or flame cleaning, with loose rust removed	C	0.30
Surfaces as rolled	D	0.20

**Table 3:** Faying surfaces according to RCSC (5.4).

Surface Treatment	Class	*µ*
(a) Unpainted clean mill scale steel surfaces (b) Surfaces with Class A coatings on blast-cleaned steel or hot-dipped (c) Galvanized and roughened surfaces	A	0.30
(a) Unpainted blast-cleaned steel surfaces (b) Surfaces with Class B coatings on blast-cleaned steel	B	0.50

**Table 4:** Average thicknesses of galvanized coating.

Tests Numbers	Average Thickness (µm)	Standard Deviation (µm)
96	97.7	14.3

**Table 5:** Series of tests.

Serie	Bolt	Tests (n)	Surface	FT Cycles	Code ^1^
14	M16 (10.9)	3	GB + ZN	No	RT
24	M16 (10.9)	3	GB + ZN	Yes	FT + RT
34	M16 (10.9)	3	GB + ZN	No	LT
44	M16 (10.9)	3	GB + ZN	Yes	FT + LT
54	M20 (10.9)	3	GB + ZN	No	RT
64	M20 (10.9)	3	GB + ZN	Yes	FT + RT
74	M20 (10.9)	3	GB + ZN	No	LT
84	M20 (10.9)	3	GB + ZN	Yes	FT + LT

^1^ RT: Slip test at Room Temperature (+20 ± 1 °C); LT: Slip test at Low Temperature (−20 ± 0.5 °C); FT + RT: Freeze Thaw Cycles and Slip test at RT; FT + LT: Freeze Thaw Cycles and Slip test at LT.

**Table 6:** Slip Factors for M16 and M20 joints.

Spec ID	RT			FT + RT			LT			FT + LT		
	*F_i_* (kN)	*F_Si_* (kN) [SD]	*µ_i_*	*F_i_* (kN)	*F_Si_* (kN) [SD]	*µ_i_*	*F_i_* (kN)	*F_Si_* (kN) [SD]	*µ_i_*	*F_i_* (kN)	*F_Si_* (kN) [SD]	*µ_i_*
M16												
1 2 3	100.15 93.33 85.62	93.03 [7.27]	0.4232	100.40 104.27 79.65	94.77 [13.24]	0.4312 (+1.87%)	134.45 104.53 111.30	116.76 [15.69]	0.5312 (+25.51%)	130.83 137.18 121.18	129.73 [8.05]	0.5902 (+39.45%)
M20												
1 2 3	200.83 199.22 191.94	197.33 [4.74]	0.5753	206.80 234.55 221.23	220.86 [13.88]	0.6439 (+11.92%)	234.70 210.25 228.22	224.39 [12.67]	0.6542 (+13.71%)	203.21 239.39 194.18	212.26 [23.93]	0.6188 (+7.56%)

## Data Availability

The authors confirm that the contributions presented in this study are available within the article. The original contributions presented in the study are included in the article. For further inquiries, please contact the corresponding author.

## References

[1] (2011). Eurocode 3: Design of Steel Structures—Part 1–8: Design of Joins.

[2] (2017). Atmospheric Icing of Structures.

[3] (2013). Eurocode 3: Design of Steel Structures—Part 1–10: Material. Toughness and Through-Thickness Properties.

[4] (2013). Eurocode 3: Design of Steel Structures—Part 3–2: Towers, Masts and Chimneys—Chimneys.

[5] (2003). Systematic Calculation of Highly Stressed Bolted Joints—Part 1.

[6] Ebert A., Dörre M., Glienke R. (2017). Behaviour of Lockbolts in Slip-Resistant Connections for Steel Structures. Steel Constr..

[7] (2018). Execution of Steel Structures and Aluminium Structures Part 2: Technical Requirements for Steel Structures.

[8] (2008). Execution of Steel Structures and Aluminium Structures Part 2: Technical Requirements for Steel Structures.

[9] Fuente-Garcia A., Serrano-López M.A., Lopez-Colina C., Lopez-Gayarre F. (2023). Slip-Resistant Bolted Connections under Freeze-Thaw Cycles and Low Temperature. Steel Compos. Struct..

[10] Fuente-Garcia A., Serrano-López M.A., Lopez-Colina C., Lopez-Gayarre F. (2024). Slip-Resistant Connections Subjected to Freeze-Thaw Cycles. Steel Constr..

[11] (2015). High-Strength Structural Bolting Assemblies for Preloading. Part 1: General Requirements.

[12] (2015). High-Strength Structural Bolting Assemblies for Preloading. Part 4: System HV Hexagon Bolt and Nut Assemblies.

[13] RCSC (2020). Specification for Structural Joints Using High-Strength Bolts.

[14] (2020). Steel Structures Australia Standard.

[15] (2018). Japanese Architectural Standard Specification: Structural Steelwork Specification for Building Construction.

[16] (1970). The Use of High Strength Friction Grip Bolts in Structural Steelwork: Metric Series Part 1: General Grade.

[17] Stranghöner N., Makevičius L., Henkel K.-M., Glienke R., Dörre M. (2021). Loss of Preload in Preloaded Bolted Connections over the Service Life. Ce/Papers.

[18] Stranghöner N., Jungbluth D., Abraham C. (2021). Weathering Impacts on the Tightening Behaviour of HV/HR-Bolting Assemblies for Preloading. Ce/Papers.

[19] Stranghöner N., Afzali N., de Vries P., Glienke R., Ebert A. (2017). Optimization of the Test Procedure for Slip Factor Tests According to EN 1090-2. Steel Constr..

[20] (2012). Cast Shot and Grit Size Specifications for Peening and Cleaning.

[21] (2015). Rust Grade Booklet.

[22] (2008). Preparation of Steel Substrates before Application of Paints and Related Products—Visual Assessment of Surface Cleanliness—Part 1: Rust Grades and Preparation Grades of Uncoated Steel Substrates and of Steel Substrates After Overall.

[23] (2023). Hot Dip Galvanized Coatings on Fabricated Iron and Steel Articles Specifications and Test Methods.

[24] (2008). Ensayos de Hormigón Endurecido. Parte 9: Resistencia Al Hielo-Deshielo: Pérdida de Masa Superficial.

[25] Charsetad H., Khorsandijou S.M. (2012). Effect of Surface Roughness on Steel-Steel Dry Friction Coefficient. Mech. Res. Appl..

[26] Chowdhury M., Helali M. (2008). The Effect of Relative Humidity and Roughness on the Friction Coefficient under Horizontal Vibration. Open Mech. Eng. J..

[27] Loganina V. (2020). Methods for Estimating Ice Adhesion to Surface. IOP Conf. Ser. Mater. Sci. Eng..

[28] Makkonen L. (2012). Ice Adhesion—Theory, Measurements and Countermeasures. J. Adhes. Sci. Technol..

[29] Stendardo L., Gastaldo G., Budinger M., Pommier-Budinger V., Tagliaro I., Ibáñez-Ibáñez P.F., Antonini C. (2023). Reframing Ice Adhesion Mechanisms on a Solid Surface. Appl. Surf. Sci..

[30] Emelyanenko K.A., Emelyanenko A.M., Boinovich L.B. (2020). Water and Ice Adhesion to Solid Surfaces: Common and Specific, the Impact of Temperature and Surface Wettability. Coatings.

[31] Bleszynski M., Clark E. (2021). Current Ice Adhesion Testing Methods and the Need for a Standard: A Concise Review. Standards.

